# Mechanism of Schwann cells in diabetic peripheral neuropathy: A review

**DOI:** 10.1097/MD.0000000000032653

**Published:** 2023-01-06

**Authors:** Jingjing Li, Ruiqian Guan, Limin Pan

**Affiliations:** a Heilongjiang University of Traditional Chinese Medicine, Harbin, Heilongjiang Province, China; b Second Affiliated Hospital of Heilongjiang University of Traditional Chinese Medicine, Harbin, Heilongjiang Province, China; c The First Affiliated Hospital of Heilongjiang University of Traditional Chinese Medicine, Harbin, Heilongjiang Province, China.

**Keywords:** diabetic peripheral neuropathy (DPN), overview, SCs

## Abstract

Diabetic peripheral neuropathy (DPN) is the most common neuropathy in the world, mainly manifested as bilateral symmetry numbness, pain or paresthesia, with a high rate of disability and mortality. Schwann cells (SCs), derived from neural ridge cells, are the largest number of glial cells in the peripheral nervous system, and play an important role in DPN. Studies have found that SCs are closely related to the pathogenesis of DPN, such as oxidative stress, endoplasmic reticulum stress, inflammation, impaired neurotrophic support and dyslipidemia. This article reviews the mechanism of SCs in DPN.

## 1. Introduction

Diabetes is a global epidemic affecting about 463 million people worldwide and is expected to affect >700 million people by 2045.^[1]^ Diabetic peripheral neuropathy (DPN) is one of the most common complications of diabetes, with a prevalence of 30 to 50% in diabetic patients,^[2]^ characterized by myelin defects, impaired nerve conduction velocity, sensory and proprioceptive abnormalities, and associated with axonal atrophy and diminished regenerative potential, ultimately leading to problems such as nerve fiber loss, pain, foot ulceration, and amputation.^[3]^ Schwann cells (SCs) are the most abundant cells in peripheral nerve tissue and play an important role in the pathogenesis of DPN. However, most of the clinical and basic studies of diabetic neuropathy focus on the effects on neurons, and there are few studies on the mechanism of SCs in DPN. Therefore, we conducted a literature search in PubMed and CNKI electronic database from November to December 2022 according to the title and abstract, and the keywords used included “diabetic peripheral neuropathy” and “Schwann cell,” as well as “diabetic peripheral neuropathy” and “Schwann cell apoptosis.” Studies related to the mechanism of action of SCs in DPN were included, and the mechanism of action of SCs in DPN was reviewed. To help understand and identify more effective treatment strategies for DPN.

### 1.1. The structure and function of SCs

SCs are the major glial cells in the peripheral nervous system. SCs can be divided into myelinated cells and non-myelinated cells according to the way they interact with axons. Myelinated nerve fibers are composed of a single axon and sheathed SCs. While unmyelinated nerve fibers have multiple axons wrapped by a single Schwann cell. These 2 types of cells are linked to axons during development through physical support and the release of various neurotrophic factors and signaling molecules. SCs have the ability of immunomodulation, inflammation and regeneration. As the main component of peripheral nerve, SCs maintain the homeostasis of the peripheral nervous system and regulate neuronal function and repair. After nerve injury, SCs around the injured axon will immediately secrete several neurotrophic factors, which bind to the corresponding receptors in the axon membrane and produce pathological signals. Pathological signals are transmitted retrogradely to the cell body and induce gene expression. In addition to providing chemical mediators, SCs themselves are directly involved in axonal regeneration. To this end, SCs undergo a series of transformation processes, including dedifferentiation, proliferation, and redifferentiation. Thus, the role of SCs in axonal function is critical under physiological and pathological conditions, leading to increasing attempts to prevent Schwann cell dysfunction or to supply SCs for the treatment of peripheral nerve diseases.^[4,5]^

### 1.2. SCs and DPN

Recent studies have found that SCs are involved in multiple pathways of DPN development. During DPN, some key signaling pathways in SCs are activated, such as high polyalcohol pathway flux driven by hyperglycemia, oxidative stress, mitochondrial dysfunction, dyslipidemia, and inflammation. Activation of these pathways and subsequent transcriptional changes lead to sustained increases in glycolysis, reactive oxygen species (ROS) formation, cellular nicotinamide adenine dinucleotide consumption, and deoxyribonucleic acid (DNA) methylation alterations that induce diabetic neuropathy, ultimately leading to myelin destruction, demyelination, axonal conduction abnormalities, and impaired neuronal regeneration.^[6]^ The injury of SCs is an important factor in the development of DPN, and taking SCs as a research target can provide a new idea for the treatment of DPN.

### 1.3. SCs and oxidative stress

Oxidative stress is caused by an imbalance between the production of ROS and the antioxidant system. In the state of diabetes, hyperglycemia stimulates SCs mitochondria to produce a large number of ROS, resulting in mitochondrial DNA damage and mitochondrial gene mutation, eventually leading to the death of SCs and axonal degeneration.^[7]^ Yanzhuo Zhang et al found that the expression of microribonucleic acid (miR)-25 decreased and the expression of ROS increased in SCs of diabetic mice, and the mechanism was related to the reduced ability of miR-25 to inhibit protein kinase C phosphorylation. miR-25 reduces ROS in diabetic peripheral nerves by inhibiting phosphorylation of protein kinase C, thereby modulating neurological dysfunction.^[8]^ Aldose reductase (AR) is highly expressed by SCs in the peripheral nervous system. Under hyperglycemic conditions, AR activity increases and catalyzes the conversion of glucose (GLU) to sorbitol. The increase in sorbitol content leads to the depletion of other osmotic hormones, such as inositol and taurine. Taurine acts as an antioxidant, and its decrease leads to oxidative and nitrosative stress in SCs. Increased AR activity also causes inhibition of glutathione reductase, leading to a decrease in reduced glutathione, resulting in excessive production of free radicals and increased oxidative stress.^[9]^ SCs also express functional thyroid-stimulating hormone (TSH) receptor. TSH is an independent risk factor for DPN. Jingwen Fan et al found that elevated TSH levels increased ROS production in rat SCs (RSC96). The possible mechanism is that the functional TSH receptor palmitoylation expressed by SCs enhances the oxidative stress induced by high GLU/palmitate (PA) in RSC96 cells.^[10]^

### 1.4. SCs and endoplasmic reticulum (ER) stress (ERS)

SCs must produce abundant myelin proteins through the ER to assemble and maintain the myelin structure. ERS impairs the synthesis of SCs myelin, and actively myelinated SCs are more susceptible to ERS than mature SCs. Lin-12-like suppressor/enhancer deficiency in SCs leads to ERS, while inactivation of the pancreatic ER kinase branch also leads to enhanced ERS and apoptosis of mature SCs in SCs-specific suppressor/enhancer-deficient mice.^[11]^ ERS of SCs can be induced by high GLU, inflammation and lipotoxicity. Palmitic acid and GLU play a synergistic role in inducing ERS-related apoptosis of RSC96. RuiLi et al evaluated the expression level of ERS-related proteins in SCs under high GLU conditions in vitro, and cultured SCs in 30 mM high GLU. The expression levels of the ERS-related proteins GLU-regulated protein-78, X-box binding protein-1 (XBP-1), activating transcription factor-6, activating transcription factor-4, and CAAT/enhanced binding protein homologous protein (CHOP) were significantly upregulated after 24 hours. The activity of SCs decreased, and the above trends were significantly reversed after treatment, indicating that ERS mediated by high GLU is very important in the injury of SCs.^[12]^ After SCs were treated with PA or PA +  GLU for 24 hours, the phosphorylation levels of GLU-regulated protein-78, XBP-1, CHOP and eukaryotic initiation factor 2α increased, most of the cells became small, round and floating, and the apoptotic events increased 100% significantly. These results suggest that GLU and PA may inhibit SCS proliferation and promote SCS apoptosis by inducing ERS in RSC96 cells.^[11]^ Keisuke Sato et al found that glycolaldehyde (GA) may induce ERS of SCs. When RSC96 were treated with 500 μM GA for 24 hours, it was observed that GA increased the number of early apoptotic cells and late apoptotic cells in a time-dependent manner. At the same time, pancreatic ER kinase, eukaryotic initiation factor 2α, and ER inositol-requiring enzyme 1α were phosphorylated, and the protein and messenger ribonucleic acid levels of CHOP were also increased. These results suggest that ERS induced by GA may trigger the apoptosis of SCs.^[13]^

### 1.5. SCs and inflammatory reaction

SCs are closely related to the inflammatory reaction. Under the condition of high GLU, the levels of tumor necrosis factor-α (TNF-α), interleukin (IL)-6, IL-1β, nuclear factor-κB (NF-κB) and Toll-like receptors in SCs were significantly increased. It may lead to axonal degeneration and axon-SCs contact disorder. Toll-like receptors and receptors for advanced glycation end products (AGE) expressed by SCs can bind to modified low-density lipoproteins and drive inflammatory responses.^[6]^ ShiqingXu et al found that when SCs were treated with different concentrations of AGEs, the oxidative stress level in cells increased after AGEs combined with receptors for AGE in SCs, NF-κB was activated and secreted various cytokines, thus causing inflammatory response and inducing apoptosis of SCs. The key factor in the injury of SCs may be the inflammation caused by AGEs.^[14]^ NF-κB can also initiate the activation of NOD-like receptor protein 3 (NLRP3) inflammasome by inducing the expression of IL-1β precursor and NLRP3. Yu-Chi Cheng et al found that the viability of RSC96 cells cultured in vitro under high GLU conditions was lost, and the expression of NF-κB-P2RX7-TNXIP was increased. NLRP3 inflammasome activation, IL-1β and IL-18 maturation, and ultimately inflammatory programmed cell death; NLRP3 inflammasome activation and apoptosis can be inhibited by loganin, which has anti-inflammatory and antioxidant effects. NLRP3 may be a new target for DPN therapy.^[15]^ Interferon-γ is also involved in the damage of SCs. WeiDu et al showed that interferon-γ could reduce the proliferation of SCs in DPN, and its mechanism was related to the inhibition of mammalian target of rapamycin complex (mTORC) 1 signaling pathway, the reduction of Rab11 expression, and the further downregulation of GLU transporter 1. Overexpression of Rab11 can reverse the decreased cell survival and proliferation induced by IFN-γ. The mTORC1 signaling pathway may mediate the expression of Rab11, a circulating endosome-associated protein, in IFN-γ-damaged SCs. Therefore, mTORC1/Rab11/GLU transporter 1 cascade pathway is an effective way to improve the function of SCs and prevent the onset of DPN.^[16]^

### 1.6. SCs and impaired neurotrophic support

SCs secrete a variety of neurotrophic factors, which play an important role in the interaction of axons and neurons with SCs. Insufficient secretion of neurotrophic factors in SCs is a very important reason for diabetic neuropathy. Animal experiments showed that the expression of brain-derived neurotrophic factor (BDNF) in the sciatic nerve of diabetic mice was decreased compared with that of non-diabetic mice. Consistent with this, hyperglycemia in mouse SCs cultured in vitro also downregulated BDNF expression by mechanisms related to inhibition of dephosphorylation of the protein kinase B (Akt)/mTOR pathway, DNA methyltransferase 1 overexpression, and DNA hypermethylation of the BDNF promoter^[17]^; trichostatin A can increase BDNF expression to improve DPN by improving the XBP-1s/ activating transcription factor-6/GRP78 axis in SCs.^[18]^ Nerve growth factor (NGF) plays a key role in nerve regeneration in the peripheral nervous system. Insufficient secretion of NGF from SCs leads to a decrease in neurite outgrowth of dorsal root ganglion neurons. 1,25(OH)2D3 can increase the ability of SCs to secrete NGF. In vitro experiments have shown that high GLU weakens the 1. 25(OH)2D3 increases the ability of SCs to secrete NGF, and the upregulation of the cytochrome P450 family member 24A1 gene plays an important role in the attenuation of the ability of 1,25(OH)2D3 to increase NGF secretion by SCs induced by high GLU.^[19]^ Other studies have found that neurin may be a potential new neurotrophic factor expressed in SCs, and the downregulation of neurin in SCs can reduce the survival rate of SCs. Moreover, exogenous neurin can improve the survival rate of diabetic SCs by increasing the level of B-cell lymphoma-2 and inhibiting the activity of caspase-3. It can also improve the neurite outgrowth of neurons in diabetic SCs co-culture. If neuroprotein metabolism is fully understood, neuroprotein supplementation can be used for the actual in vivo treatment of diabetic neuropathy.^[20]^

### 1.7. SCs and dyslipidemia

A high rate of GLU metabolism through the polyol pathway leads to dysregulation of lipid metabolism in SCs, and it is not fully understood that the accumulation of triglycerides, cholesterol, and free fatty acids in the plasma of diabetic patients appears to drive lipid-mediated neuropathology through mechanisms of oxidative and inflammatory pathways in SCs. The disruption of mitochondria in SCs leads to a shift in lipid metabolism from fatty acid synthesis to lipid oxidation, causing early depletion of the lipid components of myelin and accumulation of acylcarnitine lipid intermediates, resulting in axonal degeneration and neuropathy.^[6]^ Lipin1 is an enzyme closely related to GLU and lipid metabolism. Lipin1 expression was downregulated in DPN rats, and hyperglycemia also decreased Lipin1 expression in RSC96 cells in vitro. Downregulation of lipin1 expression prevents the synthesis of diacylglycerol, which further leads to lipid metabolism disorders and enhanced autophagy. Excessive autophagy leads to increased apoptosis of SCs and exacerbates diabetic neuropathy. Overexpression of lipin1 attenuates autophagy dysfunction and improves DPN. Lipin1 may be a potential target for the future treatment of DPN by improving autophagy disorders.^[21]^ Plasma levels of deoxysphingolipid, a newly discovered sphingolipid, are higher in high-density lipoprotein 2 in diabetic patients with neuropathy compared to patients without neuropathy. Deoxysphingolipid specifically stimulated the secretion of matrix metalloproteinase-1 and inhibited the secretion of tissue inhibitor of metalloproteinase-1 of SCs, which significantly reduced the viability of SCs and induced cytotoxicity.^[22]^ Sterol response element binding protein (SREBP)-1 is a key transcription factor that regulates lipid metabolism in a variety of tissues, and observations in diabetic rats have shown that decreased expression of SREBP-1c in SCs is accompanied by decreased MNCV. Phosphatidylinositol 3-kinase/Akt signaling pathway blockade mediates the downregulation of SREBP-1 expression by high GLU. Therefore, activating the phosphatidylinositol 3-kinase/Akt pathway or overexpressing SREBP-1 in SCs may be an effective way to treat DPN by improving lipid content.^[23]^

### 1.8. SCs and autophagy dysfunction

Autophagy is a dynamic process that maintains cellular homeostasis by recycling damaged organelles and unfolded proteins, and is considered as a protective response to maintain normal cell growth and function under physiological or pathological conditions, especially stress and metabolic disorders.^[24,25]^ High GLU is the main feature of DM, which affects the autophagy of various types of tissues and cells, such as brain, retina, renal tubular cells, podocytes and cardiac myocytes. The inhibition of autophagy has also been found in peripheral nerves of diabetic models and SCs cultured in high GLU in vitro.^[26,27]^ Zhong et al reported that LC3 expression was significantly reduced in SRY-box10 positive cells (a marker of SCs) in sciatic nerves of diabetic mice compared with non-diabetic mice. The ratio of microtubule-associated protein light chain 3 (LC3-II)/LC3-I was decreased in the sciatic nerve of diabetic mice compared with normal mice. Qu et al also showed that under high GLU conditions, autophagosomes in immortalized RSC96 cells and primary rat SCs were less than those under control conditions. Wei Du et al found that LC3-II/LC3-I and P62 were also significantly reduced in rat RSC96 cells treated with high GLU compared to normal GLU treated cells, and that LC3-II/LC3-I in RSC96 cells treated with high GLU was significantly improved by trichostatin A, but had no effect on P62 expression. Their results suggest that hyperglycemia inhibits LC3-II/LC3-I in an histone deacetylase 1-Atg3-dependent manner, and reduce P62 expression in a histone deacetylase-independent manner through the Janus kinase-signal transducer and activator of transcription proteins signaling pathway in DPN SCs.^[28]^ Modern pharmacological studies have shown that Tangbikang medicated serum can significantly increase the proliferation of SCs, upregulate the expression of yeast Atg6 homolog (Beclin1) and LC3II, which are the key molecules of autophagy, promote autophagy of RSC cells in high GLU environment, and reduce apoptosis.^[29]^ These studies suggest that the prevention of DPN by improving autophagy is a promising treatment.

## 2. Discussion

**Figure 1.**
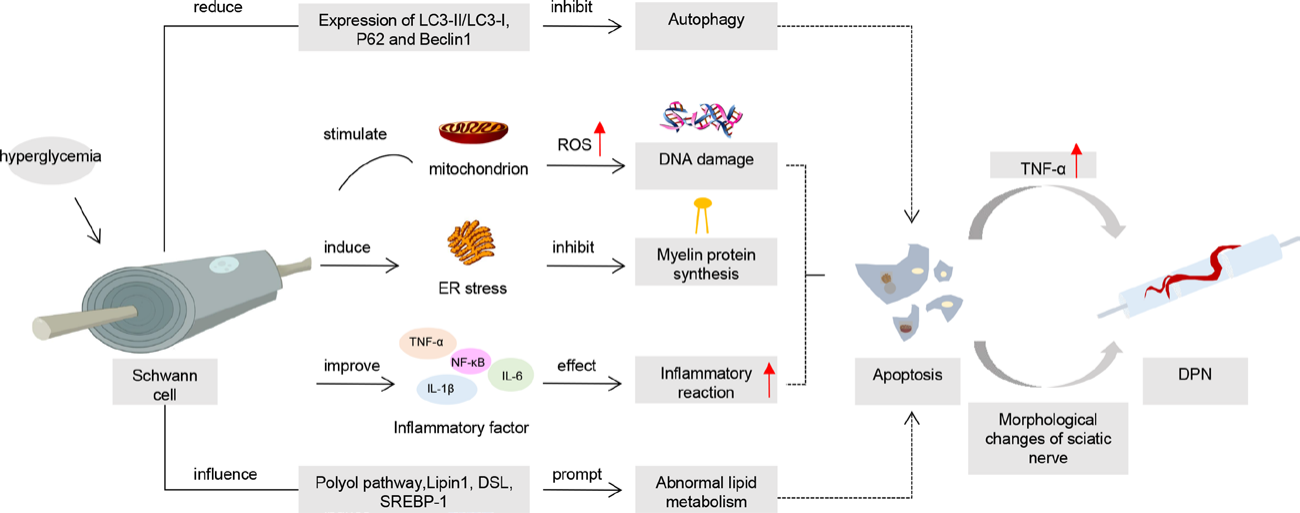
Diagram of the role of Schwan cells in DPN. DPN = diabetic peripheral neuropathy.

The pathogenesis of DPN is complex. As the most important myelin cells in the peripheral nervous system, SCs can maintain the structure and function of neurons, nourish axons, and promote survival and growth after injury. As shown in Figure 1, SCs are closely related to the pathogenesis of DPN. The apoptosis of SCs induced by hyperglycemia is involved in the pathogenesis of DPN, including oxidative stress, inflammatory response, autophagy dysfunction, ERS and other pathological processes. Leung L et al found that apoptosis of SCs can increase the expression of TNF-α, and TNF-α, as a pro-inflammatory cytokine, plays an important role in the production and maintenance of pain,^[30]^ suggesting that apoptosis of SCs may participate in the development of DPN by upregulating the expression of inflammatory cytokines. Rui Li et al found that GFs aggregate can counteract the morphological changes of sciatic nerve related to diabetic neuropathy, including improving the unclear boundary of myelin sheath and reducing the loss of nerve fibers, and effectively promote the recovery of motor and sensory functions in DPN rats. Inhibiting the apoptosis of SCs is the molecular mechanism of the protective effect of GFs aggregators on DPN. It is suggested that apoptosis of SCs may be involved in the development of DPN by destroying nerve fibers, myelin sheath boundaries, and affecting motor and sensory functions.^[31]^ The pathogenesy of DPN is still not clear. The study of the pathological process of SCs apoptosis under high GLU and the effect of apoptosis on DPN can be an important entry point for the study of DPN. It has a very positive effect on the pathogenesis of DPN, the treatment of clinical drugs, and the research and development of targeted drugs.

## Author contributions

**Conceptualization:** Jingjing Li.

**Data curation:** Jingjing Li, Ruiqian Guan, Limin Pan.

**Formal analysis:** Jingjing Li.

**Funding acquisition:** Jingjing Li.

**Investigation:** Jingjing Li.

**Methodology:** Jingjing Li.

**Project administration:** Jingjing Li.

**Resources:** Jingjing Li.

**Software:** Jingjing Li.

**Supervision:** Jingjing Li.

**Validation:** Jingjing Li.

**Visualization:** Jingjing Li.

**Writing – original draft:** Jingjing Li.

**Writing – review & editing:** Jingjing Li.
